# Hypochlorous Acid Solution Is Safe for Intracavitary Lavage: Examination in a Rodent Model

**Published:** 2021-01-09

**Authors:** Robert L. Ball, Gaurav Garg, Juan Sebastian Vazquez, Anna Day, Lauren T. Moffatt, Martin C. Robson, Jeffrey W. Shupp

**Affiliations:** ^a^Firefighters’ Burn and Surgical Research Laboratory, MedStar Health Research Institute, Washington, DC; ^b^The Burn Center, Department of Surgery, MedStar Washington Hospital Center, Washington, DC; ^c^Oak Ridge Institute for Science and Education via Department of Energy, US Army Center for Environmental Health Research, USAMRMC, Fort Detrick, MD; ^d^Department of Biochemistry and Molecular and Cellular Biology, Georgetown University School of Medicine, Washington, DC; ^e^Department of Surgery, Georgetown University School of Medicine, Washington, DC; ^f^Department of Surgery, University of South Florida, Tampa, FL

**Keywords:** hypochlorous acid, lavage, intracavitary, washout, Dakin's solution

## Abstract

Background: Intracavitary irrigation is a routine component of many surgical procedures, especially in those involving a contaminated field. Normal saline remains the irrigant of choice for most surgeons. Hypochlorous acid is a weak acid that produces hypochlorite ions with antimicrobial properties. Reducing microbial concentration during intracavitary irrigation is a potential benefit of using hypochlorous acid solution over normal saline. In this study, the safety of hypochlorous acid solution for intracavitary lavage was compared with normal saline in a rat model of 3 surgical procedures—laminectomy, thoracotomy, and laparotomy. Methods: The intracavitary space was lavaged with either normal saline or hypochlorous acid. The procedures were also completed using Dakin's solution (sodium hypochlorite) as a comparator, given its known cytotoxicity. On postoperative day 5, necropsies of all animals were performed and relevant organs and blood samples obtained. Histology (hematoxylin and eosin staining) was used to examine biopsies of the collected organs for signs of inflammation, blood vessel integrity, and necrosis. Immunohistochemistry staining for caspase-3 was used to identify apoptotic cells. Results: There were no differences in outcomes (survival, pain, and time to recovery) or histology between animals lavaged with hypochlorous acid and normal saline. Intact organ-specific architecture was observed in both groups. In comparison, rats treated with Dakin's solution demonstrated significant capsular fibrosis and hemorrhage. Furthermore, significant apoptosis was noted within the bowel mesentery of the group treated with Dakin's solution when stained for caspase-3. Conclusion: Hypochlorous acid is safe for lavage of intraperitoneal, intrathecal, and intrathoracic cavities. Further studies should be conducted to demonstrate efficacy of hypochlorous acid in an infected field.

## BACKGROUND

Intracavitary irrigation is a routine component of many surgical procedures, especially in those involving a contaminated field. Although various lavage solutions have been proposed and utilized, normal saline (NS) remains the irrigant of choice for most surgeons as it is widely available and noncytotoxic. Despite this ubiquity, there is a lack of strong evidence either for the efficacy of NS lavage or for the safety of proposed alternatives.^1^

Hypochlorous acid (HOCl) is a weak acid that produces hypochlorite ions (OCl^−^) in a solution with water. HOCl and OCl^−^ are oxidizing agents with antimicrobial properties. The ability to further reduce microbial concentration during intracavitary irrigation is a potential benefit of using a HOCl solution over NS.^2^^,^^3^ The superiority of HOCl irrigation over NS in decreasing bacterial concentration in open wounds has been previously published.^4^ In vitro studies have also demonstrated that HOCl is relatively nontoxic when compared with other commercially available antiseptics.^5^ In this study, the safety of HOCl for intracavitary lavage was compared with NS in a rat model of 3 surgical procedures—laminectomy, thoracotomy, and laparotomy.

## METHODS

Per a protocol approved by the Institutional Animal Care and Use Committee, male Sprague-Dawley rats were grouped to undergo one of the 3 surgical procedures (n = 9 for each procedure). These groups were further divided by the irrigant used for intracavitary lavage during the procedure: NS, HOCl, or half-strength sodium hypochlorite solution (NaOCl; Dakin's solution). Given its known cytotoxicity, NaOCl was used as an additional comparator to demonstrate organ injury from an irrigant.

### General procedure details

Animals were anesthetized via intraperitoneal injection of the ketamine/xylazine/acepromazine mixture: 40 mg/kg, 8 mg/kg, and 4 mg/kg, respectively.^6^ After ensuring adequate anesthesia, the animal was transferred to the operative field and placed on an oxygen nose cone. Clippers were used to remove hair from the surgical field. Procedure-specific details are described in the following sections. All 3 procedures ended with skin closure using interrupted nonabsorbable sutures. Following the procedure, 0.05 mg/kg of buprenorphine was administered for analgesia and animals were closely monitored until recovery.

### Laparotomy

**Figure 1:**
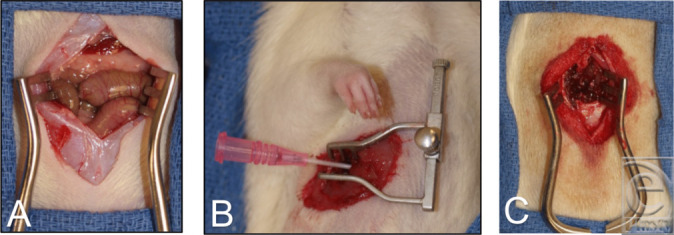
Intraoperative images of (*a*) laparotomy, (*b*) thoracotomy, and (*c*) laminectomy procedures.

A ventral midline incision was made to expose the peritoneum. The peritoneum was lifted and incised with scissors to enter the abdominal cavity. The incision was then extended to expose all intra-abdominal organs (Fig 1A). The abdominal cavity was then irrigated with 5 mL of the selected solution, which was left to instill for 2 minutes. The solution was then carefully aspirated from dependent areas of the abdomen with a syringe and an 18-gauge catheter. Suctioning near organs was avoided to limit iatrogenic injury. Irrigation and aspiration were then repeated with an additional 5 mL of solution. Before the final interrupted skin closure, the peritoneum was closed with a running nonabsorbable suture.

### Thoracotomy

Under adequate general anesthesia, animals were intubated with a 16-gauge catheter as an endotracheal (ET) tube over a blunted spinal needle that was attached to a small animal ventilator (RoVent Jr, Kent Scientific Corporation, Torrington, CT). Placement was confirmed by observation of chest rise. For the entirety of the procedure, animals were ventilated on room air with weight-based tidal volumes. A 1- to 2-cm incision was made in the skin of the right posterior-lateral chest at approximately the right fourth and fifth ribs. The intercostal space was then incised above the fifth rib to expose the thoracic cavity. A self-retaining microsurgical retractor was introduced between the ribs and opened slowly until direct visualization of the lung expanding was obtained (Fig 1B).

Through this opening, the thoracic cavity was irrigated with 3 mL of the selected solution using a 16-gauge catheter on a 5-mL syringe. After instilling for 2 minutes, easily accessible fluid was carefully aspirated while avoiding direct suction on lung tissue. This process was repeated with an additional 3 mL of solution. After the second lavage, the syringe was disconnected from the catheter that remained in the thoracic cavity. The muscle surrounding the thoracotomy was then reapproximated, and a purse string suture was placed around the catheter. An empty 5-mL syringe was then placed on the catheter. To mitigate postoperative pneumothorax, gentle aspiration was applied to the syringe as the catheter was removed while cinching the purse string. The ET tube was removed after the animal demonstrated normal respiration while disconnected from the ventilator.

### Laminectomy

With the animal in the prone position, a 2- to 4-cm incision was made over the L5-L6 spinous processes. The paraspinous muscles were then dissected free, and a small rongeur was used to remove the most accessible spinal process. Ligamentum flavum was then removed to expose the spinal cord (Fig 1C). Using a micropipette, 50 µL of the selected solution was applied and allowed to instill for 5 seconds. Any remaining accessible fluid was then aspirated off again with the pipette. The supraspinal ligament and the surrounding muscle were then reapproximated with a nonabsorbable running suture before the interrupted skin closure.

### Euthanasia and necropsy

Following the procedures, animals were monitored for surgical site infections and general systemic changes. On postoperative day 5, the animals were euthanized under inhaled isoflurane anesthesia. Animals that did not survive until day 5 were necropsied at the time of death. Surgical sites were opened, and procedure-specific organs were collected and formalin-fixed as described in Table 1.

### Histology

Hematoxylin and eosin (H&E) staining was performed on formalin-fixed organs. In addition, apoptosis was measured with immunohistochemistry (IHC) using a primary anti-caspase-3 antibody and a secondary fluorescent antibody. Images were analyzed using ZEN software from ZEISS Microscopy (Carl Zeiss Microscopy, LLC, White Plains, NY).

## RESULTS

### Morbidity and mortality

All animals survived until postoperative day 5 except for 2 of the 3 rats in the NaOCl thoracotomy group. One of these animals developed an increased work of breathing approximately 2 hours after the ET tube was removed and was unable to recover, while the other was unable to breathe independently off the ventilator at the end of the procedure. There was no appreciable pneumothorax in either animal on necropsy, but the lungs of both animals were impressively inflamed compared with lungs of NS- and HOCl-treated animals.

There were no differences in pain or behavior between the NS and HOCl groups as determined by grimace scores and observations of behavior and eating habits. For all 3 procedures, animals treated with NS or HOCl appeared to return to baseline health and activity within 6 hours after surgery. All animals treated with NaOCl, however, demonstrated increased pain and lethargy and decreased appetite that required treatment with additional fluid resuscitation and analgesia.

**Figure 2:**
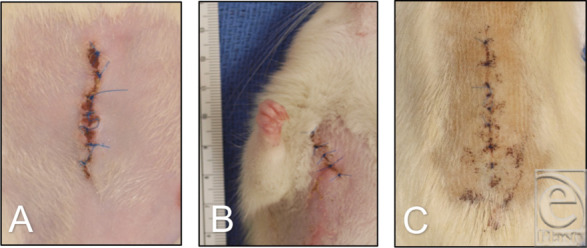
Representative images of surgical scars of (*a*) laparotomy, (*b*) thoracotomy, and (*c*) laminectomy on day 5.

**Figure 3:**
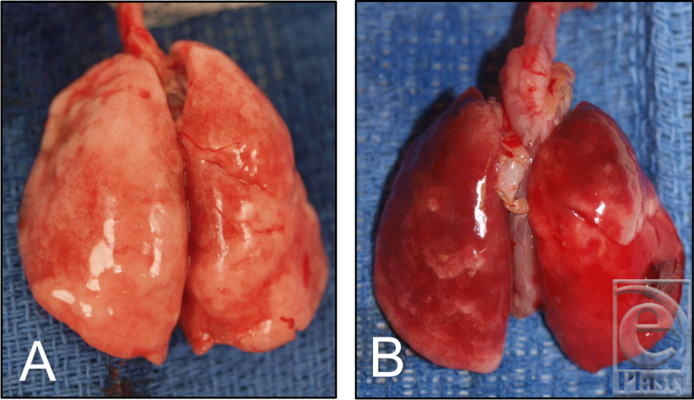
Gross specimens of lungs treated with (*a*) HOCl solution and (*b*) NaOCl solution. NaOCl-treated lungs demonstrated increased fibrosis and hemorrhage compared with NS and HOCl. HOCl indicates hypochlorous acid; NaOCl, sodium hypochlorite or Dakin's solution; NS, normal saline.

On necropsy, none of the animals were found to have developed a surgical site infection regardless of the irrigant used (Fig 2). Organs of NS- and HOCl-treated animals were grossly similar, while inflammation marked by erythema and fibrosis was noted in the lungs of all 3 NaOCl thoracotomy animals (Fig 3) and intra-abdominal organs of 2 of 3 NaOCl laparotomy animals.

### Histology

**Figure 4:**
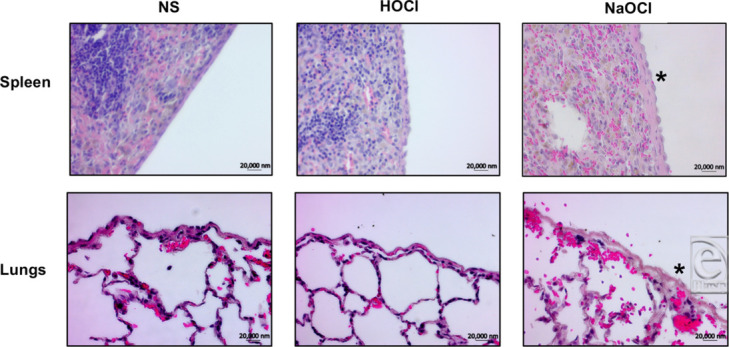
Representative H&E images of spleen (*top* row) and lung (*bottom* row) capsules exposed to the 3 different irrigants. Increased fibrosis and underlying hemorrhage was seen in the NaOCl-treated tissues (*). H&E indicates hematoxylin and eosin; NS, normal saline; HOCl, hypochlorous acid; NaOCl, sodium hypochlorite or Dakin's solution.

There were no differences on H&E staining between animals treated with NS and HOCl. Structural integrity of all tissues examined in these groups was maintained without evidence of inflammation or fibrosis. In contrast, significant inflammation, fibrosis, and hemorrhage were seen on the surfaces of tissues exposed to NaOCl lavage (Fig 4).

**Figure 5:**
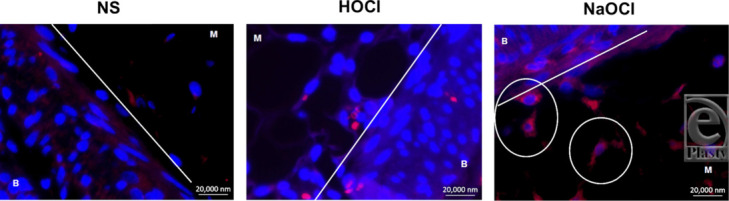
Representative images of IHC from (B) bowel wall and (M) mesentery. No apoptotic cells were seen in the NS or HOCl groups. These cells were noticeably present in the mesentery of NaOCl-treated animals (circled). IHC indicates immunohistochemistry; NS, normal saline; HOCl, hypochlorous acid; NaOCl, sodium hypochlorite or Dakin's solution.

Similar findings were found on IHC regarding the presence of apoptotic cells. The fluorescent secondary antibody identified caspase-3 in the cytoplasm of cells undergoing apoptosis. The surfaces of NaOCl-treated tissues, however, consistently demonstrated an increased presence of apoptosis compared with NS and HOCl. Apoptotic cells were especially prominent in the small bowel mesentery and lungs of NaOCl-treated animals (Fig 5).

## DISCUSSION

The study was limited by the small sample size in each group but given the stability of animals treated with NS and HOCl, it is unlikely that increasing the number will reveal significant safety differences. Also, the volume of irrigant and time to instill were limited in the laminectomy procedure in this protocol to prevent significant neurological events. Since the NS and HOCl groups did not have significant issues, these variables could be increased in future research.

The results of the study demonstrate that pain, behavior, wound healing, and organ function are not worsened by using HOCl solution as an irrigant instead of NS. The observations of animals treated with NaOCl in this study were significant as they demonstrated the systemic and histological effects of using a cytotoxic irrigant. While further research is needed to compare efficacy, the use of HOCl solution as an intracavitary irrigant was as safe as NS in this animal model of laparotomy, thoracotomy, and laminectomy. The potential benefits of an antimicrobial lavage could be observed by incorporating HOCl solution into subsequent controlled models of intra-abdominal fecal contamination, empyema, or central nervous system infection.

## CONCLUSIONS

NS, HOCl, and NaOCl were used to lavage body cavities of rats during laparotomy, thoracotomy, and laminectomy. HOCl solution was as safe as NS for lavage. Further study is needed to demonstrate the efficacy of HOCl in reducing bacterial concentration in a contaminated field.

## Figures and Tables

**Figure:**
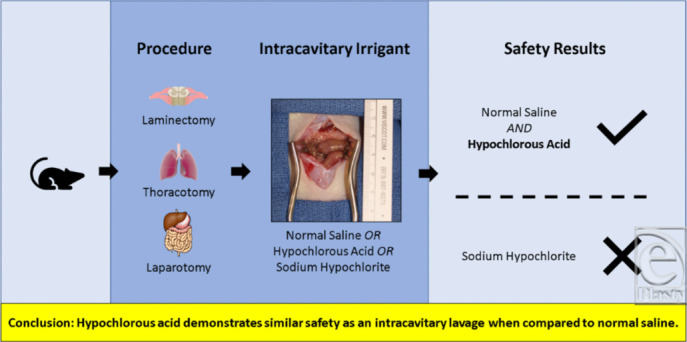


**Table 1:** Samples preserved following necropsy

Procedure	Tissue samples
Laparotomy	Liver, spleen, small bowel with mesentery
Thoracotomy	Lung, pleura
Laminectomy	Section of the spinal column including the spinal cord
